# Green Nanotechnology: Plant-Mediated Nanoparticle Synthesis and Application

**DOI:** 10.3390/nano12040673

**Published:** 2022-02-17

**Authors:** Faryad Khan, Mohammad Shariq, Mohd Asif, Mansoor Ahmad Siddiqui, Pieter Malan, Faheem Ahmad

**Affiliations:** 1 Department of Botany, Aligarh Muslim University, Aligarh 202002, India; khanfaryadamu@gmail.com (F.K.); ansarishariq.amu@gmail.com (M.S.); mansoor_bot@yahoo.co.in (M.A.S.); 2 Regional Ayurveda Research Institute, CCRAS, Ranikhet 263645, India; asifgc2616@gmail.com; 3 Unit for Environmental Sciences and Management, Mafikeng Campus, North-West University, Mmabatho 2735, South Africa; pieter.malan@nwu.ac.za

**Keywords:** biosynthesis, eco-friendly, green chemistry, nanoparticle, plant extract, sustainable application

## Abstract

The key pathways for synthesizing nanoparticles are physical and chemical, usually expensive and possibly hazardous to the environment. In the recent past, the evaluation of green chemistry or biological techniques for synthesizing metal nanoparticles from plant extracts has drawn the attention of many researchers. The literature on the green production of nanoparticles using various metals (i.e., gold, silver, zinc, titanium and palladium) and plant extracts is discussed in this study. The generalized mechanism of nanoparticle synthesis involves reduction, stabilization, nucleation, aggregation and capping, followed by characterization. During biosynthesis, major difficulties often faced in maintaining the structure, size and yield of particles can be solved by monitoring the development parameters such as temperature, pH and reaction period. To establish a widely accepted approach, researchers must first explore the actual process underlying the plant-assisted synthesis of a metal nanoparticle and its action on others. The green synthesis of NPs is gaining attention owing to its facilitation of the development of alternative, sustainable, safer, less toxic and environment-friendly approaches. Thus, green nanotechnology using plant extract opens up new possibilities for the synthesis of novel nanoparticles with the desirable characteristics required for developing biosensors, biomedicine, cosmetics and nano-biotechnology, and in electrochemical, catalytic, antibacterial, electronics, sensing and other applications.

## 1. Introduction

The nanotechnology sector has proven to be one of the most active research fields [1]. Owing to their broad uses in catalysis, sensing, electronics, photonics and medicines, the synthesis of nanoparticles has gained significant attention in recent decades [2]. Scientists have understood the potential of biological organisms to reduce metal precursors since the nineteenth century, but the mechanisms are still not known. Researchers have drawn attention towards biological methods due to the success of nanoparticle synthesis using natural reduction, capping and stabilizing agents, and avoiding harmful chemicals and high energy consumption [3,4,5]. A wide variety of products (e.g., Quantum dots (Q-dots) of cadmium sulphide, titanium oxide hybrid-based electrochemical biosensors and oxorubicin-loaded heparinized nanoparticles) can be developed through nanotechnology, and applicable to a broad array of scientific fields, including optoelectronics, biosensors, nano-biotechnology, biomedicine and others [6,7,8,9]. Creation, exploitation and synthesis are nanotechnology concepts that typically consider materials smaller than 1 mm in dimension [10]. Many different methods, such as physical, chemical and green (biological) techniques, have been used to synthesize nanoparticles [11,12,13]. The stabilized nanoparticles are formed by reducing ions through reduction (palladium NPs), nucleation (silver NPs) and growth system (silver NPs) [14,15,16]. Green chemistry, which uses chemical principles to reduce or eliminate the use of hazardous substances, has led to considerable reductions in toxic residues, which are harmful to man and the environment.

Green chemistry may be defined as chemical-assisted pollution-prevention strategies employed in specific domains such as green analytical chemistry, ecologically friendly analytical chemistry and clean analytical methodologies [17]. Thus, green synthesis is regarded as a viable approach for nanoparticle synthesis since it is biocompatible, inert and environmentally safe [18].

## 2. Different Types of Nanotechnologies

**Figure 1:**
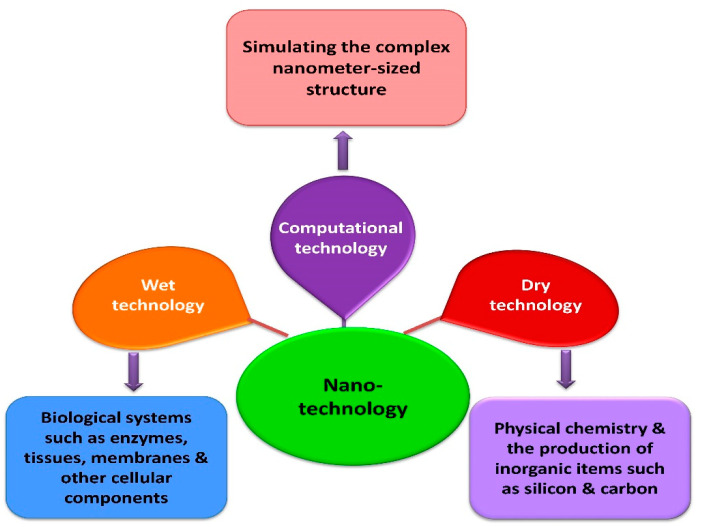
Different types of nanotechnologies.

In general, the three types of nanotechnologies are wet, dry and computational. Wet nanotechnology is concerned with the investigation of living organisms and their components such as tissues [19], enzymes and membranes [20] that are predominantly found in water-based systems [21]. Physical chemistry and inorganic compounds such as carbon and silicon are associated with dry nanotechnology. On the other hand, computational nanotechnology is associated with simulations of nanometer-sized components [22]. The three nanotechnologies, viz., wet, dry and computational, are interdependent for optimal functionality (Figure 1).

## 3. Biosynthesis of Novel Metal Nanoparticles Using Plant Extracts

**Figure 2:**
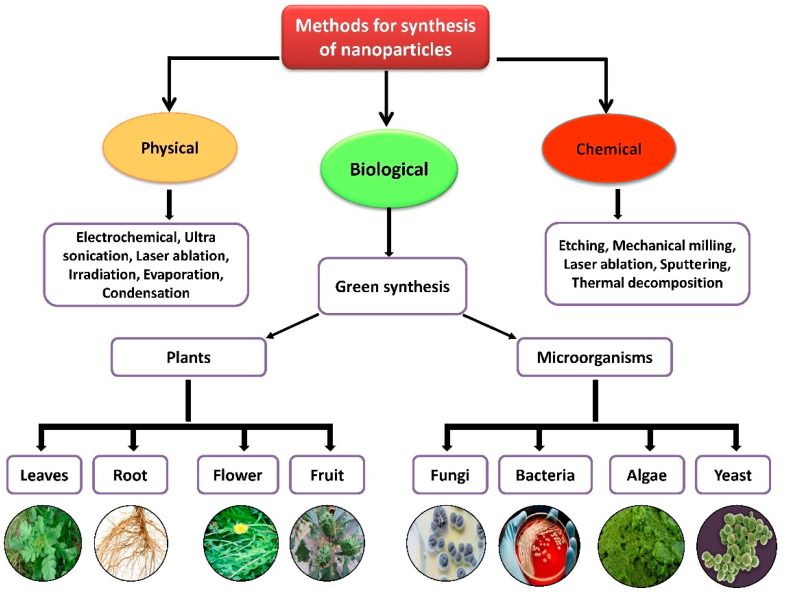
Different methods of nanoparticle synthesis.

**Figure 3:**
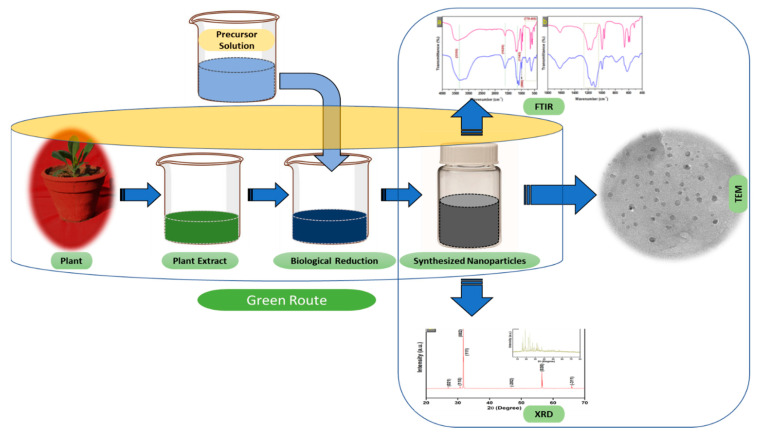
The schematic diagram for the biosynthesis of nanoparticles (NPs) via a green route using plant extract.

Nanoparticles with sizes ranging from 1 to 100 nm bind larger particles to atomic or molecular structures [23]. They are synthesized via different approaches, mainly divided into physical and chemical processes (Figure 2). The physical process involves laser ablation, condensation, evaporation, etc., whereas the chemical process involves hydrazine, sodium borohydride, green synthesis, etc. Using plant species to produce nanoparticles has been termed a green technique (Figure 2 and Figure 3) and the most reliable environmentally sustainable approach [24,25]. Nowadays, researchers are attracted towards biological synthesis, including the use of natural reducing, capping and stabilizing agents and without using hazardous, high-cost chemicals and high power consumption [26] (Figure 2 and Figure 3). NPs are extensively utilized in human contact areas (medicine, [27,28] and agriculture, [29,30]), and synthesis methods that do not use harmful compounds are increasingly required.

### 3.1. Mechanism of Nanoparticle Synthesis

**Figure 4:**
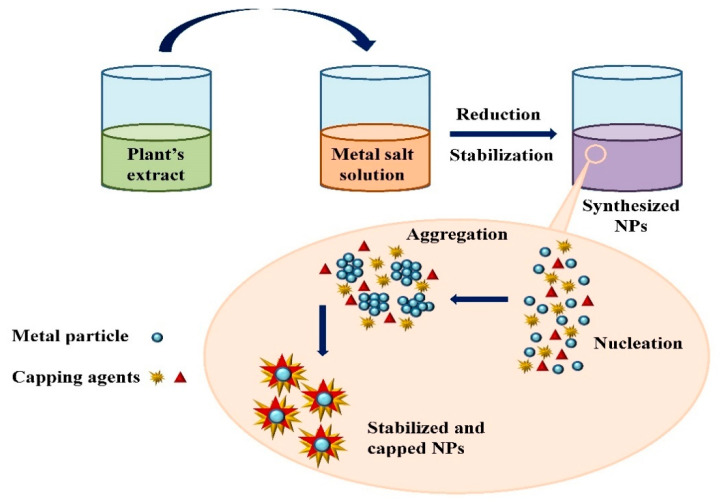
Mechanism of nanoparticle synthesis using phytoextracts.

Extensive research has been published on the testing and assessing of plants to prepare metallic nanoparticles (Figure 3), but the underlying principle for synthesizing nanomaterials has received comparatively less scientific attention [31,32]. The general tools, steps and materials involved in nanoparticle synthesis include reducing agents, capping agents, solvents, metal salts, nucleation, growth, aggregation, stabilization and characterization (Figure 4). Chemical reduction is commonly used in nanoparticle synthesis. Most methods utilize highly reactive reducing agents such as amino acids, citric acid, aldehydes, flavonoids, NADP reductase, tartaric acids, secondary metabolites, etc. Two researchers reported that the reduction potential of each metal is different and greatly affect the reduction of metals or metal precursors during synthesis. If the positive reduction potential is more, the metal precursor can be reduced at a faster rate. The nucleation and growth phases will be close to equilibrium when the reducing rate is slow [33,34]. In one-step synthesis, the slow reduction rate is also a key factor in the production of Au−Pd core–shell NPs. The finding reported the reduction potentials of PdCl_4_^2−^/Pd and AuCl^4−^/Au are 0.59 and 0.99 eV, respectively. As confirmed from the TEM analysis, during reaction the Au particles were synthesized earlier then Pd at different time intervals. This is highly consistent with PdCl_4_^2−^/Pd and AuCl^4−^/Au’s redox potential difference, and it is believed that this difference is very important for the development of the core–shell NPs [34]. In the water-soluble components of geranium leaves, Shankar et al. [35] recognized proteins and secondary metabolites. They suggested that terpenoids aid in reducing silver ions, which are then oxidized to carbonyl groups. In a study with tamarind leaf broth, the probability of an acid (tartaric acid) functional group operating as a capping medium and being essential for forming bio-reduced gold nanoparticles was studied by Ankamwar et al. [36]. This study investigated the way that alfalfa roots can absorb silver from agar media in the form of Ag(0) and transmit it to the shooting segment in the identical oxidation number [37]. The synthesized nanoparticles’ general characterization was carried out through scanning electron microscopy (SEM), transmission electron microscopy (TEM), energy-dispersive X-ray spectroscopy (EDX), ultraviolet–visible spectroscopy (UV–Vis), Fourier-transform infrared spectroscopy (FTIR) and X-ray diffraction (XRD). Microscopy (SEM and TEM) is used to determine the shape, size and particle aggregation of the desired nanoparticles without any comparison with standard materials [38]. Spectrometric techniques are the most widely used tactic for nanoparticle characterization. EDX is used to confirm the composition and distribution of the nanoparticles through spectrum and element mapping. The UV–Vis spectrometry investigates nanoparticles on the basis of particle aggregation and average particle size [39]. The basic principle of this method is absorption of plasmas by free electrons attached on the surface of nanoparticles. They interact with the electromagnetic field and shift towards higher wavelength values because the size of nanoparticles is directly proportional to higher values of wavelength. Furthermore, FTIR and XRD are applied for the determination of structural characteristics and crystallinity of formed particles.

The information on the production of various metallic NPs such as silver, gold, zinc, palladium and titanium using various plant extracts is summarized here.

### 3.2. Silver Nanoparticles

Silver nanoparticles (AgNPs) are commonly utilized nanoparticles and have attracted much study interest due to their distinctive properties. They are widely used in emerging biomedical and industrial applications [40]. AgNPs exhibit completely different characteristics from bulk materials derived from the same material due to their elevated surface/volume ratio [41]. In recent times, the synthesis of silver NPs by bio-organisms containing phytochemical agents has become an important goal for workers. Various unique secondary metabolites derived from plant extracts such as sugars, alkaloids, phenolic acids, flavonoids and terpenoids are responsible for bio-reducing ionic silver metal into nanoparticles [25,42,43].

Biosynthesis of AgNPs by *Tribulus terrestris* [44] and *Astragalus tribuloides* Delile [45] has already been reported. Spherical silver nanoparticles of size 2–6 nm were obtained from *Cycas* leaf [46]. For the synthesis of AgNPs, the affinity of *Curcuma longa* bark and powder extracts was determined. It was found that bark extract could produce more AgNPs than powder extract [47]. Kumar and Yadav [48] investigated *Lonicera japonica* plant leaf extract to develop silver and gold nanostructures. The particles obtained were different in size and shape; AgNPs were spherical to plate-like poly-shaped, and their size was 36–72 nm. Banerjee and Narendhirakannan [49] utilized seed extract of *Syzygium cumuni* to form crystalline silver nanoparticles. There is considerable data available on how to make silver nanoparticles from the latex of the *Plumeria rubra* plant [50]. Ponarulselvam et al. [51] evaluated *Catharanthus roseus* to produce silver nanoparticles because of the presence of vincristine and vinblastin. Sathishkumar et al. [52] prepared silver nanoparticles using *Cinnamomum zeylanicum* bark extract and powdered bark extract and studied the variations in the biogenic nanoparticles.

AgNPs were synthesized with a 58–458 nm range in size from the leaf extract of *Mukia maderaspatana* [53]. *Pedalium murex* was also reported to synthesize AgNPs by Anandalakshmi et al. [54]. The TEM micrographs revealed that the produced AgNPs were circular with a mean value of 50 nm. Raju et al. [55] utilized living peanut plants to synthesize AgNPs. The TEM examination showed that the biosynthesized AgNPs were of different shapes (spherical, hexagonal, triangular, square and rod-shaped) and sizes. Most of the formed AgNPs were spherical and 56 nm in average size. The EDX technique confirmed that the formed NPs were of silver. Some reports on plant-assisted synthesis of silver nanoparticles are enlisted below in Table 1.

### 3.3. Gold Nanoparticles

Gold nanoparticles (AuNPs) are the most appealing new metal NPs due to their remarkable uses in catalysis, gene expression, nonlinear optics, nanoelectronics and disease diagnostics fields [79]. Gold nanoparticles made using either phytochemicals or other extract constituents are stable for a limited period [80]. According to Sharma et al. [81], tea leaf extract can be employed in gold NP preparation. Suman et al. [82] synthesize gold NPs of size range 8–17 nm from the root extracts of *Morinda citrifolia* at ambient temperature. The biogenic production of gold nanoparticles exploiting *Nyctanthes arbortristis* alcoholic extract led to the creation of spherical-shaped nanostructures of size 19.8 ± 5.0 nm [83]. The synthesis of AuNPs was reported with Bael (*Aegle marmelos*) leaves and the particles obtained were round and 4–10 nm in size [84].

Lee et al. [38] performed the synthesis of AuNPs from the peel aqueous extract of *Garcinia mangostana*. The aqueous solution of gold in contact with *G. mangostana* extract was reduced to gold metal ions and synthesized AuNPs. The FTIR results suggested that the reducing agent found in the aqueous solution of *G. mangostana* is strongly associated with anthocyanins, benzophenones, flavonoids and phenols. The synthesized AuNPs were spherical with a size range of 32.96 ± 5.25 nm that was analyzed by TEM. Rodríguez-León et al. [85] synthesized AuNPs from the bark extract of *Mimosa tenuiflora* at different metallic (acting as precursor) concentrations.

AuNPs were made from the aqueous suspension of *Azadirachta indica* [86]. When the *A. indica* extract was mixed with Au(III) solution, the nanoparticle formation commenced. Kasthuri et al. [87] constructed gold nanoparticles with triangular and hexagonal shapes from HAuCl_4_ solution and a diluted extract possessing phyllanthin (derived from *Phyllanthus amarus*). Aromal and Philip [88] synthesized AuNPs using *Benincasa hispida* seed extract as either a reducing or capping agent. Carboxylic groups (COOH) found in the plant extract change to COO^-^ during the reduction process. The protein’s COOH group works as a surfactant, adhering to the surface of the AuNPs and then stabilizing AuNPs via electrostatic stabilization. The synthesized AuNPs were observed to have a crystalline nature and were 10–30 nm in size. Some reports on the plant-assisted synthesis of gold nanoparticles are listed below in Table 2.

### 3.4. Zinc Nanoparticles

Zinc oxide (ZnO) is an inorganic metal oxide with a vast range of nanostructures. Zinc nanoparticles (ZnNPs) have gained considerable attention due to their low cost, large surface area, white appearance, UV-filtering, antifungal, antibacterial and photochemical properties, and high catalytic activity [104,105]. There are several reports of ZnO nanoparticle synthesis using various plant extracts [106,107,108,109]. Plant extracts contain some phytochemicals (i.e., polyphenols, saponins, terpenoids) that act as reducing and stabilizing agents in the reaction system. Phytochemicals are synthesized in the plant parts, including root, stem, leaf, fruit and seed. These phytochemicals lower the metal’s valence to zero, then calcinate it to add oxide. Additionally, zinc ions interact with the polyphenols in the plant extract to form a complex. After that, zinc hydroxide (Zn(OH)_2_) is formed via hydrolysis, and then ZnO nanoparticles are synthesized after complex calculations [110].

During the literature survey, it was observed that members of the Fabaceae, Rutaceae, Apocynaceae, Solanaceae and Lamiaceae families are most commonly employed for the production of ZnNPs (Table 3). Plants from the family Lamiaceae, such as *Anisochilus carnosus*, *Plectranthus amboinicus* and *Vitex negundo* were used to produce ZnO nanoparticles of different sizes and shapes, including hexagonal, spherical, quasi-spherical and rod-shaped particles. The findings indicated that the particle sizes decrease when plant extract concentration increases [111,112]. All experiments displayed nanoparticles in the same size range with spherical and hexagonal disc shapes, which XRD and TEM analysis characterized. Singh et al. [113] synthesized ZnO NPs using *Calotropis procera* latex that were spherical and 5 nm to 40 nm in size. Ramesh et al. [114] used the floral extract of *Cassia auriculata* to react with Zn(NO_3_)_2_ solution resulting in the development of ZnNPs with a particle size ranging from 110 nm to 280 nm. Some reports on the plant-assisted synthesis of zinc nanoparticles are listed below in Table 3.

### 3.5. Titanium Nanoparticles

Titanium dioxide nanoparticles (TiNPs) have drawn great attention because of their appropriate electrical band structure, high specific surface area and quantum efficacy, stability, and chemical innerness [139]. TiNPs have a wide applicability in lowering the toxicity of synthetic dyes [140] and pharmaceutical medicines [141], wastewater treatment [142], etc. The synthesis of TiO_2_ nanoparticles on a wide scale using biological methods has stimulated the interest of researchers due to its low cost, environmental friendliness and reproducibility. Nowadays, there are many reports on the biosynthesis of TiO_2_ nanoparticles by using microbes (such as bacteria and fungi), algae, plant parts and enzymes. The aqueous extract of *Eclipta prostrata* produce nanoparticles with a spherical shape and sizes ranging from 36 nm to 68 nm, confirmed by XRD and TEM analysis [143]. Subhashini and Nachiyar [144] used the leaf extract of *Albizia saman* for the production of titanium NPs via a green route. The aqueous TiO_2_ solution was added dropwise into the leaf extract with stirring at 50 °C resulting in the formation of anatase crystals of TiO_2_ nanoparticles. The synthesized TiO_2_ nanoparticles were found to be 41 nm in size and confirmed by XRD analysis. Jalill et al. [145] synthesized the anatase form of TiO_2_ nanoparticles by using the plant extract of *Curcuma longa* (because of its terpenoid and flavonoid contents). The nanoparticles that were developed were identified by the techniques of XRD, FTIR, SEM and EDX that revealed the aggregated, circular structure and a particle size of 160–220 nm. TiNPs were synthesized by the utilization of herbal extract (as a bio-reductant) of *Echinacea purpurea* [146]. The particle size of the synthesized TiO_2_ nanoparticles was found to be in the 120 nm range. The leaf extract of *Psidium guajava* includes alcohol and primary and aromatic amines, which aid in producing TiO_2_ nanoparticles. Some reports on the plant-assisted synthesis of titanium nanoparticles are listed below in Table 4.

### 3.6. Palladium Nanoparticles

The major studies of most researchers were focused on the biological synthesis of palladium nanoparticles (PdNPs) via plant materials because it is cost-effective, sustainable, and human- and eco-friendly. Plant extracts contain a number of primary and secondary metabolites that transform metal (Pd) salts to PdNPs. Siddiqi and Husen [165] reported that the shape, size and stability of PdNPs depends on concentrations of plant extract, pH, temperature and incubation time. Plant sources including the extracts of leaves, flowers, seeds, fruits, peels and roots were extensively utilized to synthesize Pd nanoparticles.

Gurunathan et al. [166] synthesized Pd nanoparticles from a plant extract of *Evolvulus alsinoides*. This plant extract has various natural antioxidants, including alkaloids, flavonoids, saponins, tannin, steroids and phenol, which work as reducing and capping tools to synthesize Pd nanoparticles. Nasrollahzadeh et al. [167] used the leaf extract of *Hippophae rhamnoides* to synthesize PdNPs because the leaf extract has polyphenols that play an important role as reducing and capping agents for nanostructure development. The formed NPs were found to be spherically shaped and ranging from 2.5 nm to 14 nm, which was confirmed by TEM. Pd nanoparticles have been synthesized from the root extract of *Salvadora persica*, which contains polyphenols that act as reductant and stabilizing agents [168]. The average particle size of synthesized NPs was 10 nm at 90°C, which was revealed from the UV spectrum of the colloidal solution. Palladium NPs were generated with the bark extract of *Cinnamomum zeylanicum* and PdCl_2_ solution at 30 °C [169]. Khan et al. [170] carried out the plant-assisted synthesis of PdNPs from the extract of *Pulicaria glutinosa* and PdCl_2_. After stirring the mixture of PdCl_2_ + extract at 90 °C for 2 h, the colour changed from pale yellow to dark brown, indicating the production of PdNPs, validated by UV–visible spectroscopy. A TEM monograph revealed the particle size of the obtained Pd nanoparticles ranged between 20 nm and 25 nm. The particle size of the synthesized NPs was found to be between 10 nm and 50 nm. The biosynthesis of Pd nanoparticles from the leafy solution of *Glycine max* has been reported [171]. The shape of the particles was found to be uniformly spherical with a 15 nm diameter, which was confirmed by TEM micrograph. Jia et al. [172] performed the synthesis of Pd nanoparticles utilizing *Gardenia jasminoides* extract containing various antioxidants such as geniposide, crocins, crocetin and chlorogenic acid, which reduce and stabilize the nanoparticles. There are some reports on plant-assisted synthesis of palladium nanoparticles listed below in Table 5.

## 4. Factors Affecting Plant-Assisted Synthesis of Nanoparticles

**Figure 5:**
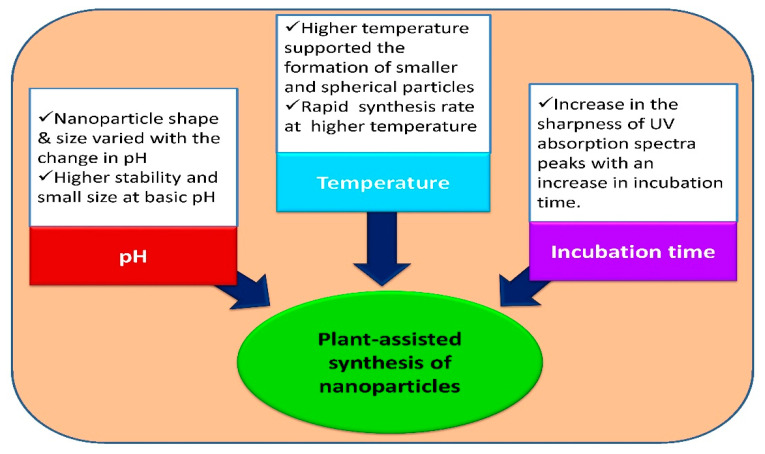
Factors affecting plant-assisted synthesis of nanoparticles.

During the biosynthesis of nanoparticles, the major difficulties often faced are maintaining the structure and size of particles in addition to obtaining mono-dispersity in the solution phase. Nevertheless, these problems can be solved by monitoring development factors, namely pH, temperature and incubation time (Figure 5).

### 4.1. Effect of pH

Several scientists have reported that pH plays a crucial role in nanoparticles’ biological synthesis. Muthu and Priya [181] studied the way that pH is an essential element for the plant-assisted preparation of silver nanoparticles and found that the size of nanoparticles increases with the decrease in pH. In this investigation the intensity of the surface plasmon resonance (SPR) peak increases with a successive rise in pH from 3 to 9 and the rate of the generation of silver NPs is greater at pH = 9. This shows the alkaline pH significantly enhances the reducing and stabilizing potential of *Ficus hispida* leaf extract in the formation of AgNPs. The number of formed silver NPs increased with higher pH because of the increased reaction rate of the leaf extract of the test plant and thus NPs with a small particle size were observed [182]. Armendariz et al. [183] stated that the size of gold NPs prepared from *Avena sativa* extract was directly pH-dependent. The experiment conducted by Zulfiqar et al. [184] reported the stability of the biosynthesized silver nanoparticle colloid at pH 4. Another study reported that alakaline pH (8) at room temperature results in the formation of diverse-shaped gold NPs from the leaf extracts of *Angelica archangelica*, *Hypericum perforatum* and *Hamamelis virginiana* with sizes ranging from 4 to 8 nm in diameter [185]. Dhamecha et al. [186] observed that red to dark purple color gold NPs were formed depending upon the pH. NPs with a purple colour were produced at pH 7, a fluorescent purple colour at pH 10 and no colour was noticed in acidic pH 2. Sathishkumar et al. [169] tested the pH effect over a broader range (1–11) in *Cinnamom zeylanicum* and bark-extract-synthesized silver nanoparticles. They found, after the synthesis of silver NPs, a drop in the pH of the solution in most cases. Dubey et al. [70] observed that AgNPs had a reduced zeta potential value (−26 mV) in highly acidic pH solutions than at alkaline pH, indicating that nanoparticles at basic pH are more stable and smaller in size. At pH 8, the colloid consists of nanoparticles of approximately 20 nm in size, with triangular, hexagonal and nearly spherical shapes. In the present study the average size of AgNPs at pH 4 was 32.7 nm and they were spherical in shape. As the pH of the reaction increased to 7, the mean size of the NPs decreased to 7.12 nm. This shows a direct relation between the pH of the extract and nanoparticle size [187]. Silva-De-Hoyos et al. [188] observed that high pH, i.e., 7.8, led to the development of AuNPs with a size of 11–20 nm.

### 4.2. Temperature Role in Plant-Assisted Synthesis

In most studies, regarding the influence of the reaction temperature, it was evaluated that the size of nanostructures is inversely proportional to the temperature. At room temperature (27 °C), NPs with a mean size of 49.91 nm and distorted spherical shape were found. As the temperature increases moderately to 45 °C, the size of silver NPs starts reducing to 33.61 nm, with a more uniform spherical shape [187]. Fayaz et al. [189] also reported that the size of the NPs decreases at higher temperatures and increases at lower temperatures. Silver nanoparticles using olive leaf extract were synthesized by Khalil et al. [190]. They found that on increasing the temperature, there was a quick reduction of Ag^+^ ions and the simultaneous uniform nucleation of silver nuclei allowing the formation of nanoparticles of a small size. At high temperatures, a higher reduction rate was observed because of the utilization of silver ions in nuclei production, whereas the secondary reduction was halted over the surface of predetermined nuclei [69]. Similarly, the intensity of the SPR peak was increased with elevation in temperature. The enhanced reaction temperature causes faster reduction of the Ag^+^ ions and successive homogeneous nucleation of Ag NPs results in production of small sized particles. When the temperature changes from 35 to 90 °C, the intensity of the SPR peak is also shifted to high. Further temperature rises above 90 °C result in decreased intensity of the SPR peak and hence 90 °C is considered as the optimum temperature for AgNP synthesis [182]. Song et al. [191] studied the role of temperature on the formation of nanoparticles. They found that a high temperature favored the formation of small and spherical particles, whereas, at a lower temperature, polydispersed particles of size 5–300 nm were extracted.

### 4.3. Contact or Incubation Role in Plant-Assisted Synthesis

Many scientists have worked on nanoparticle synthesis and showed the effect of the incubation period. Bar et al. [62] evaluated the impact of reaction time on synthesis of AgNPs using the optimized concentration of AgNO_3_ (0.005 M) and latex extract (3%) of *Jatropha curcas*. It was observed that the intensity of SPR peaks increases as the reaction time proceeds and after 4 h of incubation period, two SPR bands separated by more than 50 nm were achieved. Philip [192] suggested that in a plant-mediated synthesis, silver nanoparticles’ size was dependent on the contact time. Ghoreishi et al. [193] also documented the importance of an appropriate reaction time in the stable synthesis of gold and silver NPs with *Rosa damascena.* While dealing with *Chenopodium* leaf extract, the authors of [194] observed a clear rise in the peaks of UV absorption spectra on increasing contact time. They obtained nanoparticles within 15 min of the reaction and these kept rising for about 2 h, but a slight deviation was observed after it. Likewise, Dubey et al. [70] noted that the synthesis of Au and Ag NPs was initiated after 10 min of the reaction in Tansy fruit-mediated synthesis. The UV–Vis spectral analysis showed enhancement in the absorbance intensity of the reaction mixture with incubation time, which consequently resulted in solution stability after 24 h of exposure, indicating the successful synthesis of silver nanoparticles [195].

## 5. Application of Nanoparticles

Nanotechnology has attracted researchers’ interest because of the microscopic size and high surface-to-volume ratio of nanoparticles, which results in chemical and physical changes in the characteristics. Due to these properties, nanoparticles have a great variety of applications in several biomedical, environmental and agricultural sectors.

Hydrophilic (water-soluble) nanoparticles have been employed as drug carriers for many years. The most efficient nanoparticles used for this purpose are polyethylene oxide nanoparticles [27]. Their ability to deliver drugs in an optimum range has enhanced therapeutic efficiency and patient compliance. Among the metal nanoparticles, Au, Ag and Fe NPs have been widely used in medical applications. AuNPs are used in drug delivery, bioimaging and photothermal therapy [196], whereas AgNPs are used for drug delivery, wound dressing, cancer therapy and to restrict the growth of microbial infection [197]. ZnNPs have recently been applied as antimicrobial and anticancer agents due to their potential to generate reactive oxygen species [197], and nanoparticles synthesized using copper have also been used in a wide range of biomedical applications [198,199]. Similarly, nanoparticles of iron, gold, silver, copper, zinc and titanium are applied as antimicrobial agents to inhibit the growth of infectious bacteria and fungus and thus induce mortality [29,30].

In the environment sector, due to the ratio of surface area to mass, nanoparticles play a very important role in the purification of water through binding with precipitates, debris and heavy metals [200]. This binding depends on the composition, morphology and absorbency of the nanoparticles. Nanoparticles are applied in the field of environment in three different ways. Firstly, making environment-friendly products through green chemistry to avoid pollution [201]. Secondly, the bioremediation of environmental contaminants [202]. Thirdly, nanoparticles are used as sensors to identify changes in environmental stages [142,203]. TiO_2_ nanoparticles are an effective photocatalytic agent used in water treatment. The use of these nanoparticles to filter out the organic contaminants from several water reservoirs has been explored [204]. FeNPs have gained attention because of their potency to bioremediate heavy metals, namely lead, mercury, arsenic, cadmium and thallium from water [205]. In addition to bioremediation, photo-degradation by NiO and ZnO nanoparticles has also been accomplished [206,207]. The efficient photo-degradation was due to nano-sized nanoparticles (10–50 nm) [208].

**Figure 6:**
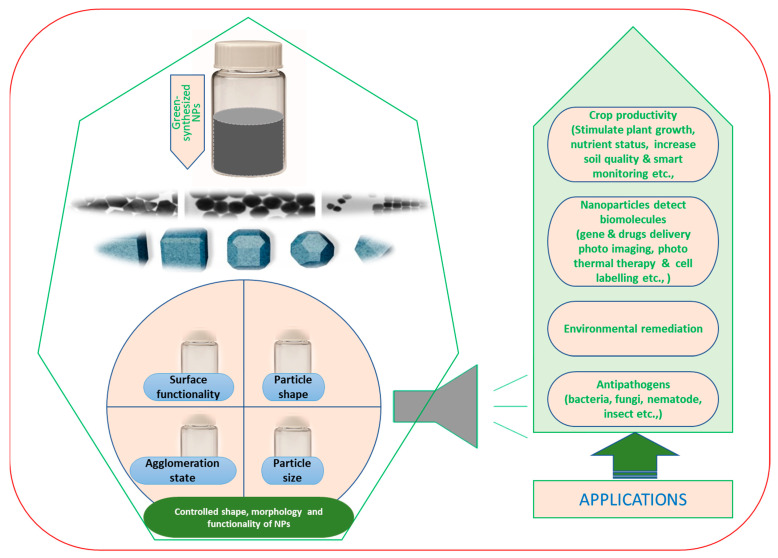
An overview diagram shows synthesized nanoparticles (NPs) produced via the green route for various biological applications. The different sizes, shapes and surface bio-functionalized NPs are developed in a controlled way for the target application.

Nanoparticles have many potential applications in the field of agriculture due to their antimicrobial activity. In the agriculture sector, nanoparticles are used as nano-formulations of agrochemicals to be applied as pesticides and fertilizers for crop improvement, nano-sensors for recognizing diseases to protect the crop and nano-devices for genetic engineering of plants. The agricultural applications of antimicrobial nanomaterials have increased since the last decade. Silver nanoparticles have been found to be very effective against *Bacillus cereus*, *Staphylococcus aureus*, *Shigella flexneri* and *Escherichia coli* [45]. Similarly, antimicrobial activity has also been reported for several other green-synthesized nanoparticles, including gold, zinc, titanium and palladium [89,109,148,176]. An overview of nanotechnology applications in agriculture is presented in Figure 6.

At present, green-synthesized metal nanoparticles are viewed as powerful nanotechnology to manage hazardous soil-borne microbes. Many green-synthesized metal nanoparticles have been explored for their antimicrobial properties. These include silver, iron, copper, silicon, silica, graphene, gold, palladium, zinc oxide, titanium dioxide, selenium oxide and carbon nanotubes. Currently, the application of green nanoparticles is being encouraged to manage plant-parasitic nematodes as they have a multisite mode of action and no phytotoxicity (Figure 6).

## 6. Conclusions and Future Roles

The traditional nanoparticle synthesis approaches are expensive and generate potentially toxic substances; it is necessary to reduce the risk of contamination from the various chemicals used during chemical and physical methods. The generation of nanoparticles using extracts of different plant species, or green synthesis, has emerged as an important front in nanotechnology. Furthermore, plant extracts are readily available to develop an efficient and healthy green route for the scale-up and industrial development of well-dispersed metallic nanoparticles.

This review emphasizes recent research findings in novel metal nanoparticle plant-assisted synthesis and critically examines the various mechanisms proposed to explain it. The plant-assisted synthesis of metal NPs derived from plant extracts has multiple positive aspects: eco-friendliness, biocompatibility and cost-effectiveness. Researchers have prioritized the investigation of the biochemical pathways and enzymatic reactions of nanomaterials biosynthesis, as well as the identification and characterization of biomolecules associated with nanoparticle synthesis. Research is an ongoing operation, with researchers from different fields regularly contributing more substantial solutions to the significant problems.

## Figures and Tables

**Table 1:** Plant-assisted synthesis of silver nanoparticles.

Plant Name	Parts Used	Size (nm)	Shapes	Reference
*Morinda citrifolia* L.	Leaves, fruit pulp, seeds	3–11	Spherical	[56]
*Nymphae odorata*	Leaves	15 ± 5	Spherical	[57]
*Capparis zeylanica*	Leaves	23	Spherical	[58]
*Caesalpinia pulcherrima*	Leaves	9	Spherical	[59]
*Carya illinoinensis*	Leaves	12–30	Spherical	[60]
*Mentha piperita*	Leaves extract	35	Spherical	[61]
*Jatropha curcas*	Latex	10–20	Face-centered cubic	[62]
*Acalypha indica*	Leaves extract	20–30	Spherical	[63]
*Hibiscus rosa sinensis*	Leaves	14	Spherical/prism	[64]
*Cycas*	Leaves	2–6	Spherical	[46]
*Ceratonia siliqua*	Leaves extract	5–40	Spherical	[65]
*Suaeda monoica*	Leaves	31	Spherical	[66]
*Catharanthtus roseus*	Leaves	35–55	Cubical	[51]
*Ocimum sanctum*	Leaves extract	10–20	Spherical	[67]
*Ocimum tenuiflorum*	Leaves	25–40	Spherical	[68]
*Ginkgo biloba*	Leaves	15–500	Cubic	[69]
*Tanacetum vulgare*	Fruit	16	Spherical	[70]
*Argemone mexicana*	Leaves extract	30	Spherical, hexagonal	[71]
*Sesuvium portulacastrum*	Callus extract	5–20	Spherical	[72]
*Syzygium cumini*	Leaves and seed	29–92	Spherical	[49,73]
*Cinnamomum camphora*	Sun dried leaves	3.2–20	Cubic hexagonal crystalline	[74]
*Melia azedarach*	Leaves	78	Spherical	[75]
*Rhododedendron dauricam*	Flower extract	25–40	Spherical	[76]
*Lippia citriodora*	Leaves extract	15–30	Crystalline	[77]
*Tribulus terrestris*	Fruit	16–28	Spherical	[44]
*Citrullusm colocynthis*	Leaves	31	Spherical	[78]

**Table 2:** Plant-assisted synthesis of gold nanoparticles.

Plant Name	Parts Used	Size (nm)	Shapes	Reference
*Parkia biglobosa*	Leaves	1–35	Truncated, pentagonal, spherical, triangular	[39]
*Curcuma pseudomontana*	Rhizome	20	Spherical	[89]
*Lawsonia inermis*	Leaves	20	Spherical	[90]
Cinnamon	Bark	35	Spherical	[91]
*Croton Caudatus Geisel*	Leaves	20	Spherical	[13]
*Tamarind*	Leaves	20–40	Triangle	[36]
*Aloe vera*	Plant extract	50/350	Crystalline	[92]
*Mentha, Ocimum, Eucalyptus*	Leaves	3–16	Spherical	[93]
*Canna indica, Quisqualis indica*	Leaves and flower	30–130	Polymorphic/stable	[94]
*Murraya koenigii*	Leaves	20	Spherical	[95]
*Aegle marmelos*	Leaves	4–10	Spherical	[84]
*Rosa hybrid*	Rose petals	10	Cubic	[96]
*Terminalia chebula*	Plant extract	6–60	Anisotropic	[97]
*Momordica charantia*	Fruit	30–40	Cubical	[98]
*Phyllanthus amarus*	Leaves	65–99	Cubic	[99]
*Mangifera indica*	Leaves	17–20	Spherical	[100]
*Stevia rebaudiana*	Leaves	8–20	Octahedral	[101]
*Nyctanthes arbortristis*	Flower extract	19.8	Spherical, hexagonal	[83]
*Trigonella foneum-graecum*	Leaves	15–25	Spherical	[79]
*Tanacetum vulgare*	Fruit	11	Triangular	[70]
*Cuminum cyminum*	Seeds	1–10	Spherical	[102]
*Sorbus aucuparia*	Leaf extract	16–18	Spherical, triangular, hexagonal	[103]

**Table 3:** Plant-assisted synthesis of zinc nanoparticles.

Plant Name	Parts Used	Size (nm)	Shapes	Reference
*Artemisia pallens*	Leaves along with stem	50–100	Hexagonal	[109]
*Cayratia pedata*	Leaves	52.24	Spherical	[115]
*Euphorbia hirta*	Leaves	20–50	Spherical	[116]
*Eucalyptus globules*	Leaves	52–70	Spherical, elongated	[108]
*Tecoma castanifolia*	Leaves	70–75	Spherical	[117]
*Zingiber officinale*	Root	30–50	Spherical	[118]
*Azadirachta indica*	Leaves	50	Spindle shaped	[119]
*Catharanthus roseus*	Leaves	23–57	Spherical	[120]
*Solanum nigrum*	Leaves	20–30	Hexagonal	[121]
*Olea europea*	Leaves	18–30	Crystalline	[122]
*Azadirachta indica*	Leaves	25	Crystalline	[123]
*Nyctanthes arbor-tristis*	Flowers	12–32	Crystalline	[124]
*Hibiscus rosa-sinensis*	Leaves	30–35	Crystal, spongy	[125]
*Ruta graveolens*	Stem	28	Spherical	[106]
*Aloe vera*	Leaves	22.18	Hexagonal	[126]
*Ocimum tenuiflorum*	Leaves	11–25	Hexagonal	[127]
*Sargassum muticum*	Leaves	30–57	Hexagonal	[128]
*Calotropis gigantea*	Leaves	1.5–8.5	Spherical	[107]
*Beta vulgaris*	Root	52–76	Hexagonal	[129]
*Curcuma longa*	Root	20–80	Hexagonal	[130]
*Nephelium lappaceum*	Peel	20	Spherical	[131]
*Artocarpus gomezianus*	Fruit	50	Spherical	[132]
*Senna auriculata*	Leaves	2	Spherical	[133]
*Brassica oleraceae*	Leaves	1–100	Spherical and sheet shaped	[134]
*Acalypha Indica*	Leaves	100–200	Cube	[135]
*Plectranthus amboinicus*	Leaves	20–50	Crystalline	[136]
*Coptidis rhizome*	Rhizome	2.9–25.2	Spherical and rod shaped	[137]
*Ginger*	Rhizome	23–26	Crystalline	[138]

**Table 4:** Plant-assisted synthesis of titanium nanoparticles.

Plant Name	Parts Used	Size (nm)	Shapes	Reference
*Ledebouria revoluta*	Bulb	47	Tetragonal	[147]
*Pouteria campechiana*	Leaves	73–140	Spherical	[148]
*Syzygium cumini*	Leaves	22	Spherical round	[149]
*Mentha arvensis*	Leaves	20–70	Spherical	[150]
*Azadirachta indica*	Leaves	15–50	Spherical	[151]
*Psidium guajava*	Leaves	32.58	Spherical	[152]
*Nyctanthes arbor-tristis*	Leaves	100–150, 100–200	Cubic, crystalline, Spherical	[153]
*Calotropis gigantea*	Flower	10–52	Crystalline, Spherical oval	[154]
*Salvia officinalis*	Leaves	15–20	Spherical	[140]
*Solanum trilobatum*	Leaves	70	Spherical, oval	[155]
*Azadirachta indica*	Leaves	124	Spherical	[156]
*Annona squamosal*	Leaves	40–60	Spherical	[157]
*Jatropha curcas, citrus aurantium*	Leaves	25–50	Spherical	[158]
*Jatropha curcas*	Latex	25–50	Spherical, uneven	[159]
*Euphorbia prostrata*	Leaves	81–84	Spherical	[160]
*Citrus sinensis*	Fruit peel	19	Tetragonal	[161]
*Cassia auriculata*	Leaves	38	Spherical	[162]
*Ocimum basilicum*	Leaves	50	Hexagonal	[163]
*Hibiscus-rosa-sinensis*	Petals	7–24	Spherical	[12]
*Erythrina variegates*	Leaves	39	Crystalline, spherical	[164]

**Table 5:** Plant-assisted synthesis of palladium nanoparticles.

Plant Name	Parts Used	Size (nm)	Shapes	Reference
*Peganum harmala*	Seed	22.5 ± 5.7	Spherical	[173]
*Coleus amboinicus*	Leaves	40–50	Spherical	[174]
*Anogeissus latifolia*	Gum ghatti	4.8 ± 1.6	Spherical	[175]
*Filicium decipiens*	Leaves	2–22	Spherical	[176]
*Cinnamomum camphora*	Leaves	3.2–6	Multiple	[177]
*Pulicariaglutinosa*	Leaves	3–5	Spherical	[170]
*Musa paradisica*	Peeled banana	50	Crystalline	[178]
*Cinnamom zeylanicum*	Bark	15–20	Crystalline	[169]
*Catharanthus roseus*	Leaves	38	Spherical	[179]
*Curcuma longa*	Tuber	10–15	Spherical	[180]
*Glycine max*	Leaves	15	Spherical	[171]

## Data Availability

Not applicable.
